# Fungal-specific IgG responses in allergic conjunctivitis: comparison with IgE and immunological implications

**DOI:** 10.7717/peerj.20625

**Published:** 2026-01-08

**Authors:** Tatsuya Mimura

**Affiliations:** 1 Department of Ophthalmology, Teikyo University School of Medicine, Itabashi-ku, Tokyo, Japan; 2 Department of Ophthalmology, Tsurumi University Shool of Dental Medicine, Yokohama, Kanagawa, Japan

**Keywords:** Allergic conjunctivitis, IgE, IgG, Fungi, *Alternaria*, *Aspergillus*, *Cladosporium*, *Penicillium*, Tear IgE, Skin Prick Test

## Abstract

**Background:**

Immunoglobulin G (IgG) is the most abundant antibody class in the bloodstream and is characterized by a long half-life compared to other immunoglobulins. While IgG plays a key role in host defense against infections, it is also known to be elevated in chronic inflammatory conditions. This study aimed to evaluate serum levels of fungus-specific Immunoglobulin E (IgE) and IgG antibodies in patients with allergic conjunctivitis and to investigate their associations with disease severity and sensitization status.

**Methods:**

A total of 40 patients with allergic conjunctivitis and 20 healthy controls were enrolled. Serum levels of specific IgE and IgG antibodies against four common fungi—*Alternaria*, *Aspergillus*, *Cladosporium*, and *Penicillium*—were measured. These values were compared with skin prick test (SPT) results and clinical severity scores for allergic conjunctivitis (0–30 scale).

**Results:**

Patients with allergic conjunctivitis exhibited significantly higher positivity rates and serum titers of both IgE and IgG against all four fungal antigens compared to controls (*p* < 0.05). Notably, IgG titers were significantly higher than IgE titers across all fungal antigens (*p* < 0.05). IgG levels demonstrated stronger correlations with SPT positivity (correlation coefficients *r* = 0.95–0.97 *vs. r* = 0.60–0.89 for IgE) and allergic conjunctivitis severity scores (*r* = 0.35–0.60 *vs. r* = 0.23–0.43 for IgE, *p* < 0.001).

**Conclusions:**

Serum fungus-specific IgG antibodies may serve as useful biomarkers reflecting the severity of allergic conjunctivitis. Given that mucosal barrier dysfunction has been implicated in allergic inflammation, the findings suggest that hypersensitivity reactions to fungal elements due to impaired barrier function may contribute to disease pathogenesis.

## Introduction

Allergic conjunctivitis is an Immunoglobulin E (IgE)-mediated hypersensitivity disorder of the conjunctival epithelium, characterized by symptoms such as itching, hyperemia, chemosis, and tearing (Tariq, 2024). Seasonal allergic conjunctivitis and perennial allergic conjunctivitis are the most prevalent subtypes, affecting approximately 20% of the global population in some form (Tariq, 2024). While IgE-driven immediate hypersensitivity reactions are the primary mechanism underlying disease onset, chronic and severe forms of allergic conjunctivitis are thought to involve more complex immune responses (Miyazaki et al., 2020; Miyazaki et al., 2022).

Immunoglobulin G (IgG), the most abundant antibody class in serum, is known for its long half-life compared to other immunoglobulins. In addition to its role in host defense against infections, elevated IgG levels are often observed in chronic inflammatory conditions. Specific IgG (sIgG) against environmental antigens has been used as a marker of exposure; for instance, sIgG against *Aspergillus* species has been reported to reflect disease severity in patients with type 2 chronic rhinosinusitis (CRS) (Hung et al., 2024). In the ophthalmic field, allergen-specific IgG has also demonstrated clinical relevance; for example, sIgG against dog allergens has been shown to significantly correlate with the severity of perennial allergic conjunctivitis associated with pet exposure (Miyama et al., 2015; Mimura, Noma & Mizota, 2019).

Fungal exposure, particularly to *Alternaria*, *Aspergillus*, *Cladosporium*, and *Penicillium*, has been implicated in chronic airway inflammation in conditions such as CRS and respiratory allergies, with corresponding increases in fungus-specific IgG levels (Shin et al., 2004). In patients with eosinophilic mucin chronic rhinosinusitis (EMCRS), fungus-specific IgG—especially the IgG3 subclass—has been found to be significantly elevated compared to healthy controls and disease controls, suggesting that IgG may be more closely associated with severe disease than IgE (Pant et al., 2005). These findings highlight the potential of IgG as a biomarker reflecting immune responses distinct from those mediated by IgE, and underscore its possible role in assessing disease severity in allergic conditions caused by indoor environmental allergens such as dust mites and fungi.

However, the role of fungus-specific IgG in allergic conjunctivitis remains poorly understood. The present study aimed to investigate the serum levels of IgE and IgG specific to four representative indoor fungi—*Alternaria*, *Aspergillus*, *Cladosporium*, and *Penicillium*—and their associations with clinical severity scores and skin prick test (SPT) reactivity. We sought to determine whether fungus-specific IgG may serve as a useful indicator of disease severity in allergic conjunctivitis.

## Materials & Methods

### Study design

**Figure 1:**
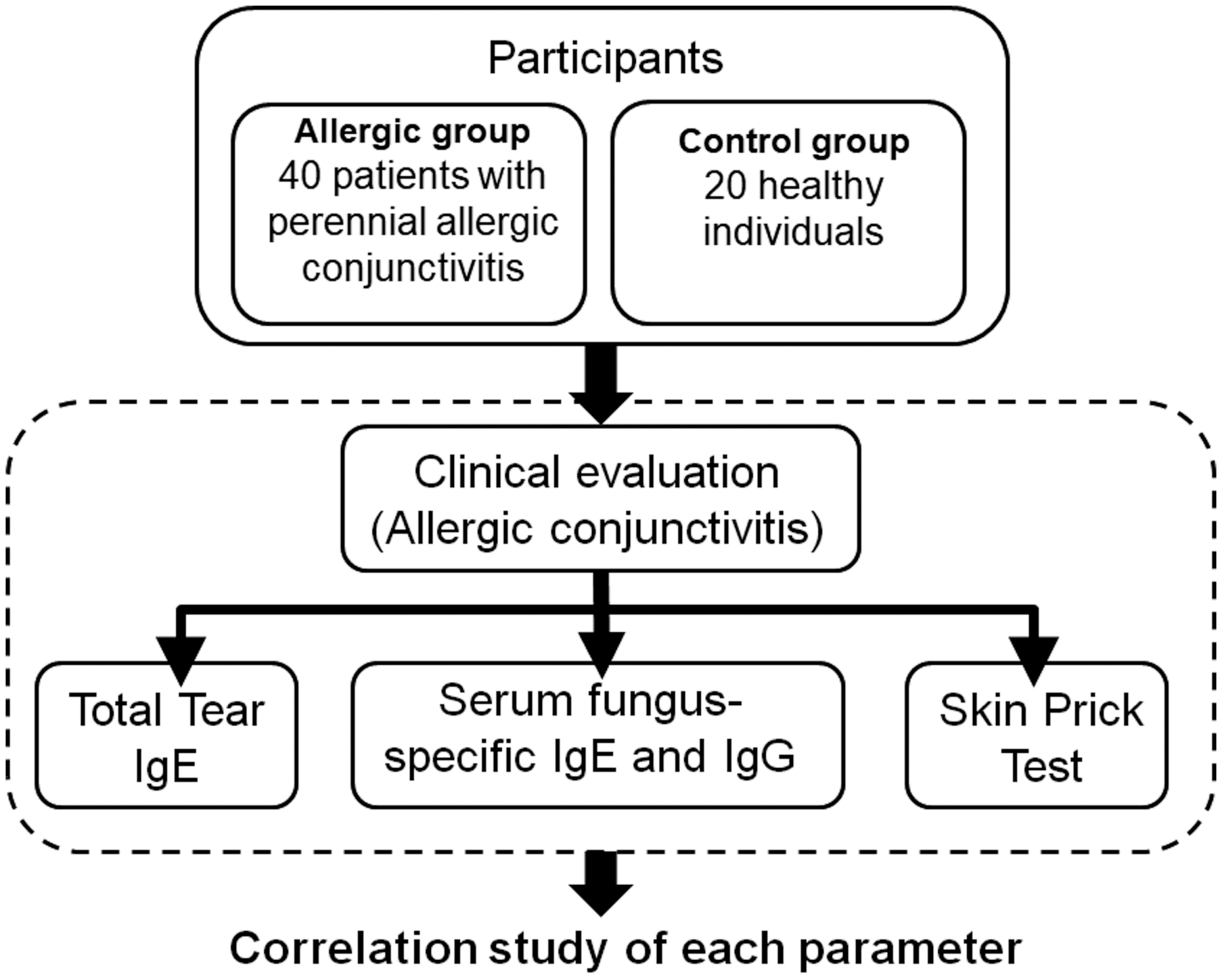
Schematic overview of the experimental protocol.

This prospective, non-randomized, cross-sectional study with consecutive patient enrollment was conducted at Teikyo University Hospital, and affiliated institutions. The study adhered to the principles outlined in the Declaration of Helsinki. The study protocol was approved by the Ethics Committees of Teikyo University School of Medicine (18-228). Several related studies, including this one, have been registered with the University Medical Information Network for Clinical Trials (UMIN-CTR, registration number: UMIN000013687). Both patient recruitment and the study were carried out from April 2020 to October 2021. Written informed consent was obtained from all participants prior to inclusion. This study did not receive any funding, sponsorship, or financial support from pharmaceutical companies or other organizations. Figure 1 presents a schematic overview of the experimental protocol.

### Participants

All participants’ medical histories were obtained through structured interviews and reviewed medical records. Inclusion criteria for patients included a diagnosis of perennial allergic conjunctivitis based on clinical history and ophthalmologic examination conducted by experienced ophthalmologists. All participants underwent a comprehensive ocular surface examination. Tear secretion was assessed using the Schirmer I test without topical anesthesia. A standardized Schirmer strip (Whatman filter paper, 5 × 35 mm) was placed in the lateral one-third of the lower eyelid margin for 5 min under ambient conditions. The length of wetting (in mm) was measured after 5 min, and a value of >10 mm was considered within the normal range. Meibomian gland function was evaluated by slit-lamp biomicroscopy. The orifices were examined for obstruction or capping, and gentle digital pressure was applied to the lower eyelid to assess meibum quality and expressibility. All participants were considered to have normal meibomian gland function when clear meibum was easily expressed from most glands without signs of obstruction, thickening, or dropout.

Patients with dry eye syndrome, atopic blepharoconjunctivitis, atopic keratoconjunctivitis, vernal keratoconjunctivitis, contact lens wearers, and those with a history of cataract surgery, corneal refractive surgery, or infectious conjunctivitis were excluded. A total of two groups of participants were enrolled in the study. The allergic group consisted of 40 patients diagnosed with perennial allergic conjunctivitis, while the control group comprised 20 healthy, age- and sex-matched non-smoking individuals with no history of allergic diseases. Control participants were recruited from patients visiting outpatient clinics for routine ophthalmic examinations.

### Data collection

A detailed allergy history was obtained from the patient through clinical interview. Demographic information was not collected, as it was beyond the scope of this report. Comprehensive ophthalmic examinations, including measurements of visual acuity and refractive status, as well as slit-lamp biomicroscopy, were performed to evaluate the ocular findings; however, these data were not included in this report because they were not directly related to the main subject of interest.

Serum samples were collected *via* venipuncture using standardized procedures and processed immediately for immunological assays. Clinical severity scores were evaluated by trained clinicians using a standardized grading scale. The skin prick test was performed following a standardized protocol with commercial allergen extracts, and positivity was defined according to established criteria. All immunological and clinical data were collected on the same day to ensure consistency.

### Diagnosis and scoring of allergic conjunctivitis

The diagnosis of allergic conjunctivitis was based on subjective symptoms such as ocular itching and tearing, and objective signs including conjunctival hyperemia, follicle formation, and papillary hypertrophy observed with slit-lamp biomicroscopy. Patients exhibited persistent conjunctival itching, redness, and tearing throughout the year, with no clear seasonal variation, and had a clinical history consistent with continuous allergen exposure. Immunological markers, including serum-specific IgE levels against common perennial allergens, supported the diagnosis. This approach helped differentiate perennial allergic conjunctivitis from other forms such as seasonal allergic conjunctivitis, which typically presents with symptoms limited to specific seasons and correlates with allergen exposure periods. Diagnostic criteria were based on previously published guidelines (Miyazaki et al., 2020; Miyazaki et al., 2022).

At the initial visit, clinical severity was assessed using a scoring system derived from the Japanese Allergic Conjunctival Disease Quality of Life Questionnaire (JACQLQ) (Fukagawa, 2012; Mimura et al., 2011). The author has permission to use this instrument from the copyright holders. The clinical evaluation consisted of 10 parameters encompassing findings from the palpebral conjunctiva, bulbar conjunctiva, limbus, and cornea. Palpebral conjunctival findings included hyperemia, edema, follicle formation, papilla formation, and the presence of giant papillae. Bulbar conjunctival findings were limited to hyperemia and edema. At the limbus, Trantas dots and edema were assessed. Corneal involvement was evaluated based on epithelial damage. Each parameter was graded on a four-point scale according to severity: 0 = normal, 1+ = mild, 2+ = moderate, and 3+ = severe. The total allergic conjunctivitis score (range: 0–30) was calculated as the sum of all parameter scores. Although both eyes were evaluated, only the data from the right eye were used for analysis to ensure consistency.

### Measurement of total tear IgE levels

The total IgE concentration in tear fluid was measured using the Allerwatch^®^ test, in accordance with the manufacturer’s instructions (Hitachi Chemical Co., Ltd., Tokyo, Japan; and Wakamoto Pharmaceutical Co., Ltd., Tokyo, Japan), as previously described by Mimura et al. (2011). The intensity of the red line that appears on the test strip corresponds to the total IgE concentration in the tear sample. No coloration is observed when the IgE concentration is within the normal range (<2.0 kU/L). Based on the visual assessment of the test line, results were classified into three grades: Grade 0, indicating undetectable IgE (no coloration of the test line); Grade 1, indicating a low IgE concentration (test line fainter than the control line); and Grade 2, indicating a high IgE concentration (test line equal to or darker than the control line). Following visual evaluation, the results were confirmed using Scion Image software (Scion Co., Frederick, MD, USA).

### Measurement of serum fungus-specific IgE and IgG antibodies

Fungus-specific IgE and IgG antibody levels in serum were quantified using an enzyme-linked immunosorbent assay (ELISA) as previously described (Miyama et al., 2015). The fungal antigens tested were *Alternaria*, *Aspergillus*, *Cladosporium*, and *Penicillium*.

In brief, fungal antigens were pre-coated onto ELISA plates. Serum samples were added and incubated to allow antigen–antibody binding. Unbound components were removed by washing, followed by the addition of HRP-conjugated anti-human IgG (or IgE) antibodies. After further washing, tetramethylbenzidine substrate was added for color development. The reaction was stopped with a stop solution, changing the color to yellow. The optical density (OD) was measured at 450 nm, and titers were calculated based on OD values. IgE titers were also quantified and scored on a 0–6 class scale.

### SPT

SPT for fungal allergens was performed using sterile disposable lancets (Yayoi Co., Ltd., Tokyo, Japan). A drop of allergen solution (1:20 wt/vol; Torii Pharmaceutical Co., Ltd., Tokyo, Japan) was applied to the forearm and introduced into the epidermis with a lancet. Physiological saline served as a negative control. Results were read after 15 min, and a mean wheal diameter >three mm was considered positive.

### Statistical analysis

Based on previous studies that found significant differences with sample sizes of 10 per group (Miyama et al., 2015; Mimura, Noma & Mizota, 2019), we aimed to recruit at least 20 participants per group. A sample size calculation with an error margin of 10%, confidence level of 90%, power of 0.8, and standard deviation of 10 indicated that 22 subjects per group would be sufficient to detect significant differences. Thus, a target of 20 subjects per group was deemed statistically appropriate. Additionally, the control group consisted of 20 healthy volunteers, which was sufficient for comparison because the variability in clinical and biochemical parameters among healthy subjects was minimal. In contrast, a larger number of patients with perennial allergic conjunctivitis (*n* = 40) were included to capture the broader range of disease severity and to improve the robustness of the statistical analyses.

Mean values were compared using a two-tailed unpaired Student’s *t*-test. Frequencies were analyzed with the chi-square test or Fisher’s exact test. For semiquantitative data, the Mann–Whitney *U* test (two groups) or Kruskal-Wallis one-way analysis of variance (ANOVA) (three or more groups) was used. Correlations between variables were assessed using Pearson’s correlation coefficient, and differences in correlation strength were tested by Fisher’s *Z* transformation. Sensitivity, specificity, and positive and negative predictive values were calculated using standard formulas. All statistical analyses were performed using SAS System software version 9.1 (SAS Institute Inc., Cary, NC, USA), with a significance threshold set at *p* < 0.05.

## Results

### Demographic and clinical characteristics

The allergic group consisted of 40 patients (14 males and 26 females; mean age, 25.2 ± 16.4 years) diagnosed with perennial allergic conjunctivitis. The control group comprised 20 healthy, non-smoking individuals (seven males and 13 females; mean age, 24.6 ± 9.0 years). There were no significant differences in age or sex distribution between the two groups. All patients in the allergic group had a history of persistent ocular itching and redness lasting for more than 12 months, and several reported a previous diagnosis of allergic rhinitis or atopic dermatitis. Detailed demographic data, such as socioeconomic status and educational background, were not collected, as they were beyond the scope of this study.

### Clinical findings of allergic conjunctivitis

**Figure 2:**
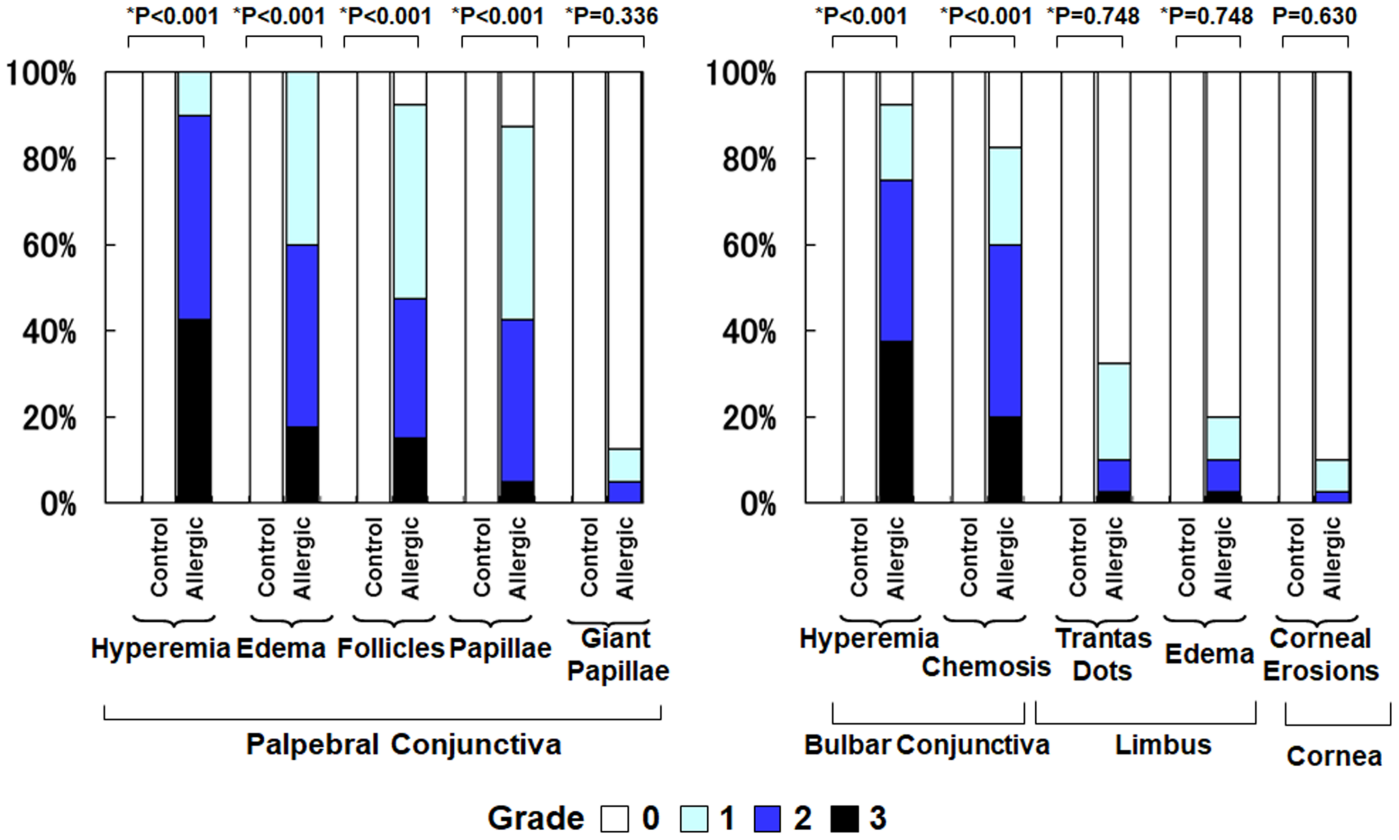
Comparison of clinical findings of allergic conjunctivitis between the allergy group and the control group. An asterisk (*) indicates that between-group comparisons were performed using the two-tailed Mann–Whitney U test.

Figure 2 compares the clinical findings between the control group and the allergic group. The allergic group showed significantly higher scores across all evaluated parameters compared to the control group (all *p* < 0.001, Mann–Whitney U test). Specifically, the mean ± standard deviation (SD) values in the control group *versus* the allergic group were as follows: palpebral conjunctival hyperemia (0.0 ± 0.0 *vs.* 2.3 ± 0.6), palpebral conjunctival edema (0.0 ± 0.0 *vs.* 1.8 ± 0.7), palpebral follicles (0.0 ± 0.0 *vs.* 1.6 ± 0.8), palpebral papillae (0.0 ± 0.0 *vs.* 1.4 ± 0.8), giant papillae (0.0 ± 0.0 *vs.* 0.2 ± 0.5), bulbar conjunctival hyperemia (0.0 ± 0.0 *vs.* 2.1 ± 0.9), bulbar conjunctival edema (0.0 ± 0.0 *vs.* 1.6 ± 1.0), Trantas dots (0.0 ± 0.0 *vs.* 0.5 ± 0.7), limbal edema (0.0 ± 0.0 *vs.* 0.3 ± 0.7), and corneal epithelial erosion (0.0 ± 0.0 *vs.* 0.1 ± 0.4).

### Total tear IgE levels

**Figure 3:**
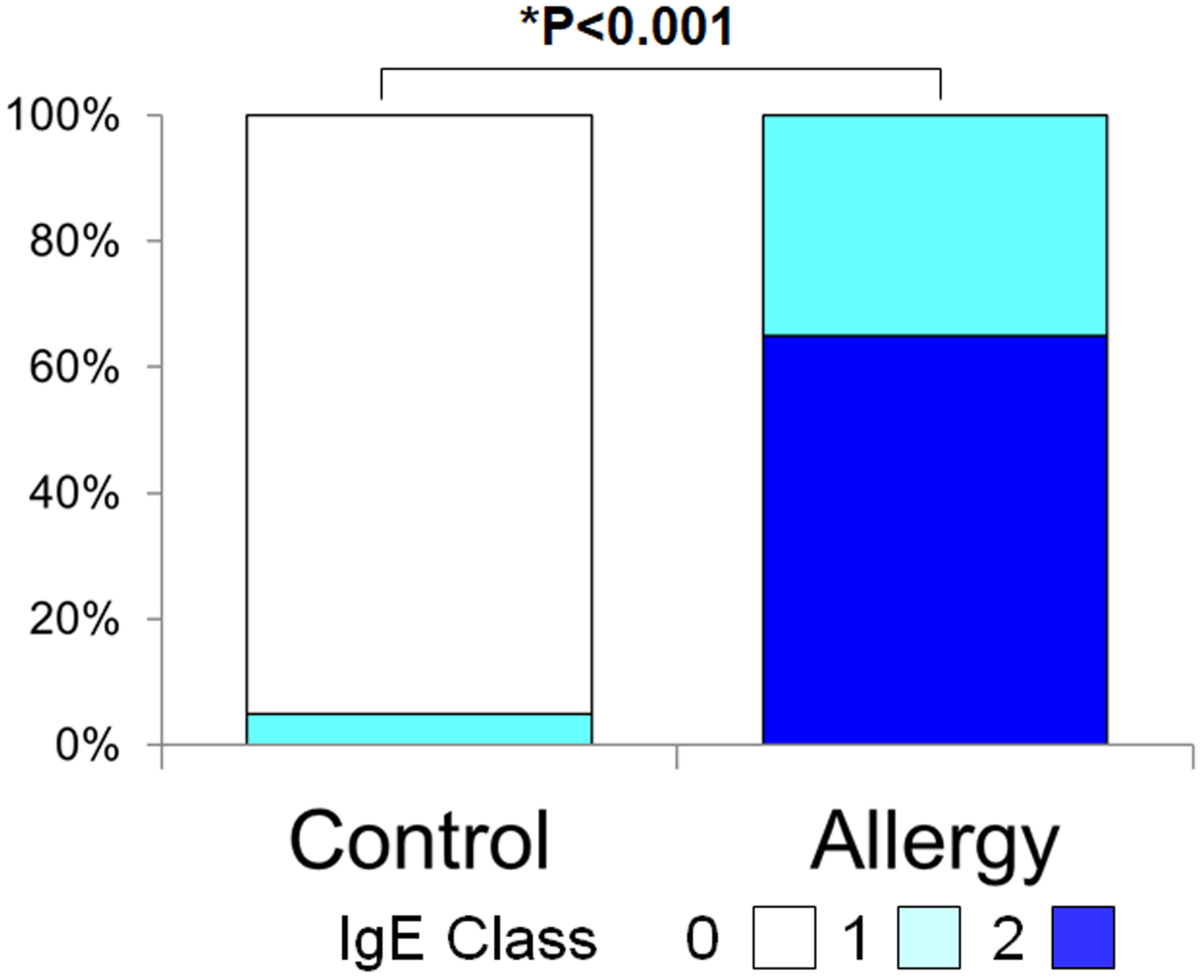
Comparison of the grade of positivity for total tear immunoglobulin E (IgE) between the allergy group and the control group. Total tear IgE was determined by the Allerwatch â test and a grade was assigned as described in Methods. An asterisk (*) indicates that results were compared between the two groups by the two-tailed Mann–Whitney U test.

Figure 3 shows the positivity rate and grading score of total tear IgE. In the allergic group, all subjects (100%) were positive, which was significantly higher than the control group (5.0%) (*p* < 0.001, Fisher’s exact test). Moreover, total tear IgE scores were significantly higher in the allergic group compared to controls (1.65 ± 0.48 *vs.* 0.05 ± 0.22, *p* < 0.001, two-tailed Mann–Whitney U test).

### Serum specific IgE to fungal allergens

**Figure 4:**
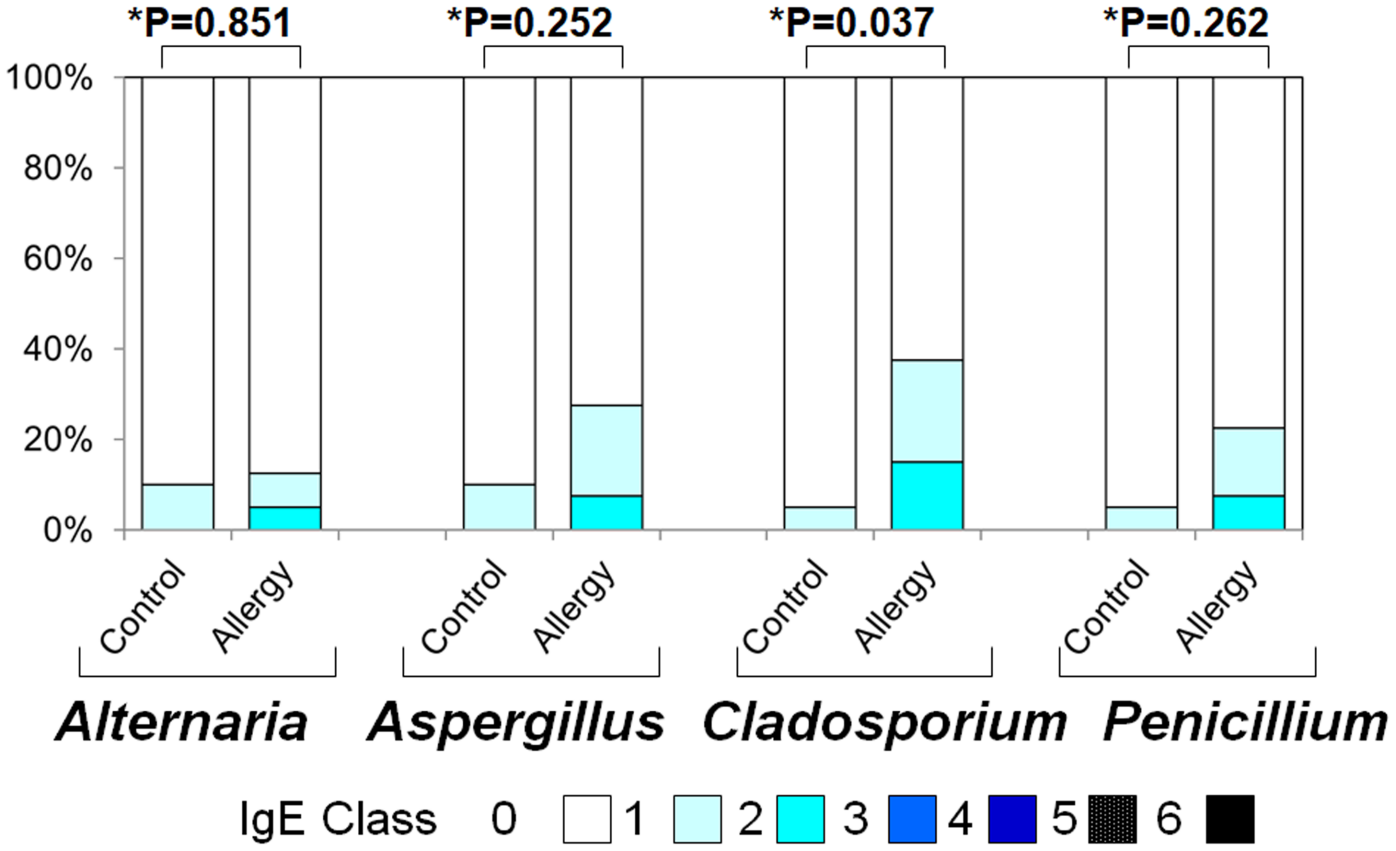
Serum specific IgE positivity rates and antibody titers against fungal allergens in the allergy and control groups. The allergy group showed higher positivity rates and antibody titers for *Alternaria, Aspergillus, Cladosporium*, and *Penicillium* compared to the control group. An asterisk (*) indicates that results were compared between the two groups by the two-tailed Mann–Whitney U test.

Figure 4 illustrates the positivity rates and serum levels of specific IgE antibodies against fungal allergens. In all four fungi (*Alternaria, Aspergillus, Cladosporium,* and *Penicillium*), both positivity rates and antibody titers were generally higher in the allergic group than in controls:

**Figure 5:**
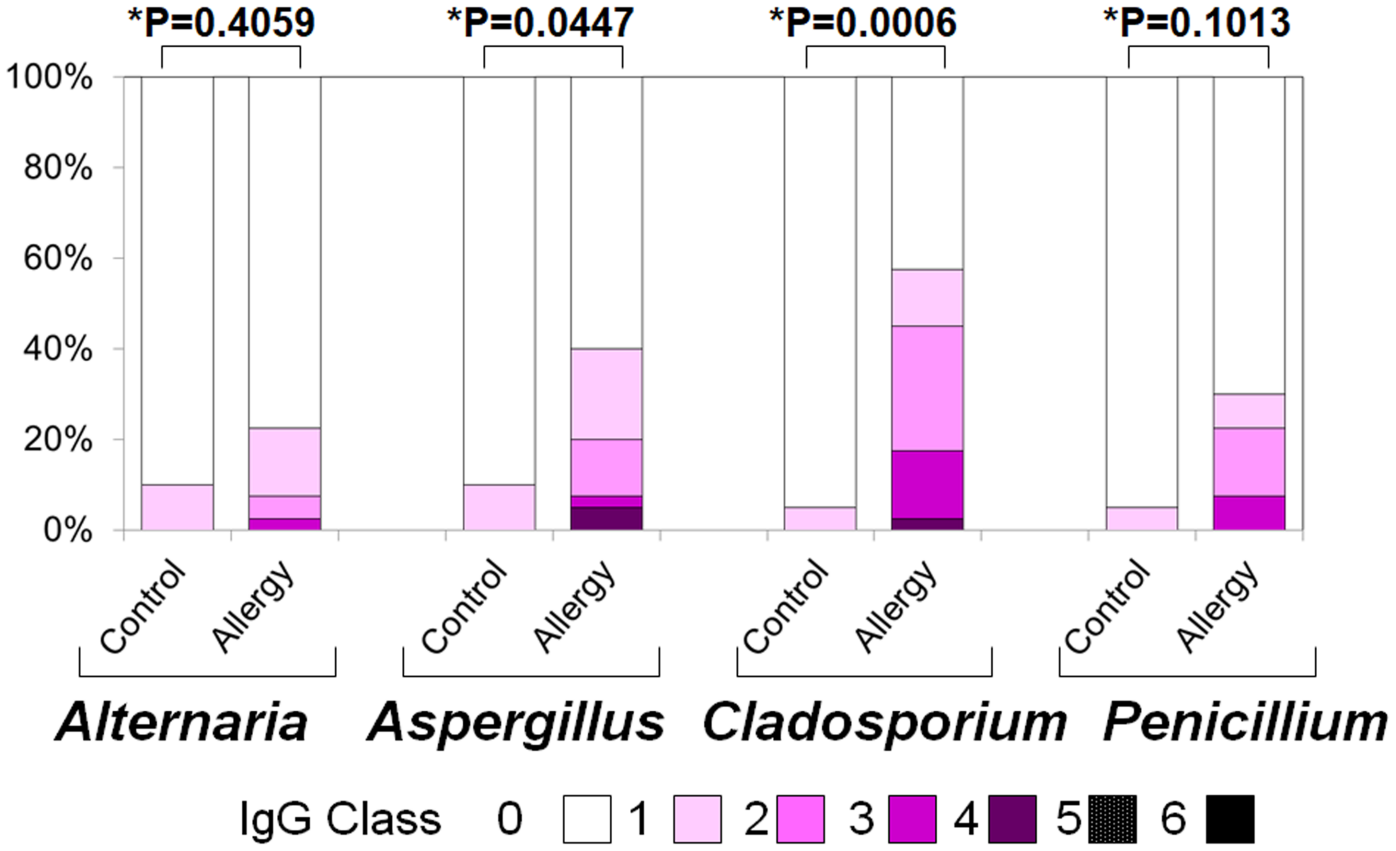
Serum specific immunoglobulin G (IgG) positivity rates and antibody titers against fungal allergens in the allergy and control groups. The allergy group showed higher positivity rates and antibody titers for *Alternaria, Aspergillus, Cladosporium*, and *Penicillium* compared to the control group. An asterisk (*) indicates that results were compared between the two groups by the two-tailed Mann–Whitney U test.

• ***Alternaria***: Positivity rate: 10.0% *vs.* 12.5% (*p* = 0.023), Antibody titer: 0.10 ± 0.30 *vs.* 0.18 ± 0.49 (*p* = 0.851)
• ***Aspergillus***: 10.0% *vs.* 27.5% (*p* < 0.001), 0.10 ± 0.30 *vs.* 0.35 ± 0.61 (*p* = 0.252)
• ***Cladosporium***: 5.0% *vs.* 37.5% (*p* < 0.001), 0.05 ± 0.22 *vs.* 0.53 ± 0.74 (*p* = 0.037)
• ***Penicillium***: 5.0% *vs.* 22.5% (*p* < 0.001, Fisher’s exact test), 0.05 ± 0.22 *vs.* 0.30 ± 0.60 (*p* = 0.262, two-tailed Mann–Whitney U test)

### Serum specific IgG to fungal allergens

Figure 5 presents the positivity rates and serum levels of specific IgG antibodies against the same fungal allergens. The allergic group exhibited higher IgG positivity rates (Fisher’s exact test) and titers (two-tailed Mann–Whitney U test) for all fungi:

• ***Alternaria***: 10.0% *vs.* 22.5% (*p* < 0.001); 0.10 ± 0.30 *vs.* 0.33 ± 0.69 (*p* = 0.406)
• ***Aspergillus***: 10.0% *vs.* 40.0% (*p* < 0.001); 0.10 ± 0.30 *vs.* 0.73 ± 1.10 (*p* = 0.045)
• ***Cladosporium***: 5.0% *vs.* 57.5% (*p* < 0.001); 0.05 ± 0.22 *vs.* 0.60 ± 0.99 (*p* < 0.001)
• ***Penicillium***: 5.0% *vs.* 30.0% (*p* < 0.001); 0.30 ± 0.78 *vs.* 2.88 ± 3.36 (*p* = 0.101)

### SPT results

**Figure 6:**
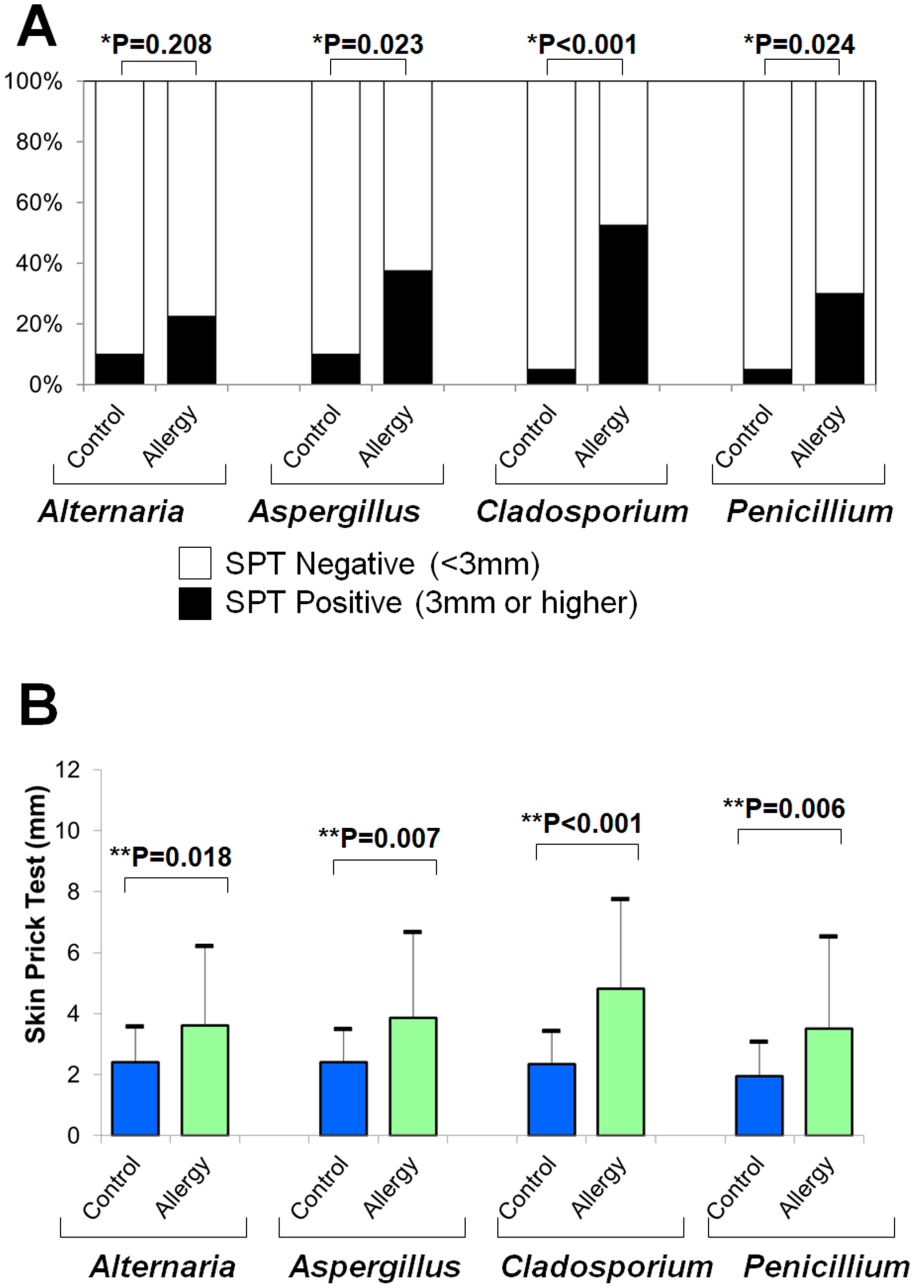
Results of skin prick tests (SPTs) for fungal allergens. (A) Positivity rates were compared between the allergy and control groups using Fisher’s exact test (*). (B) Mean wheal diameters were compared between the two groups using a two-tailed unpaired *t*-test (**).

Figure 6A shows the positivity rates of the SPT for fungal allergens. The allergic group demonstrated higher rates than controls across all four fungi (Fisher’s exact test):

• ***Alternaria***: 10.0% *vs.* 22.5% (*p* = 0.208)
• ***Aspergillus***: 10.0% *vs.* 37.5% (*p* = 0.023)
• ***Cladosporium***: 5.0% *vs.* 52.5% (*p* < 0.001)
• **Penicillium**: 5.0% *vs.* 30.0% (*p* = 0.024)

Figure 6B shows that the average wheal diameters were significantly larger in the allergic group for all fungi (two-tailed unpaired *t*-test):

• ***Alternaria***: 2.4 ± 1.2 mm *vs.* 3.6 ± 2.6 mm (*p* = 0.018)
• ***Aspergillus***: 2.4 ± 1.1 mm *vs.* 3.9 ± 2.8 mm (*p* = 0.007)
• ***Cladosporium***: 2.4 ± 1.1 mm *vs.* 4.8 ± 2.9 mm (*p* < 0.001)
• ***Penicillium***: 2.0 ± 1.1 mm *vs.* 3.5 ± 3.0 mm (*p* = 0.006)

### Correlation between SPT and serum antibodies

**Figure 7:**
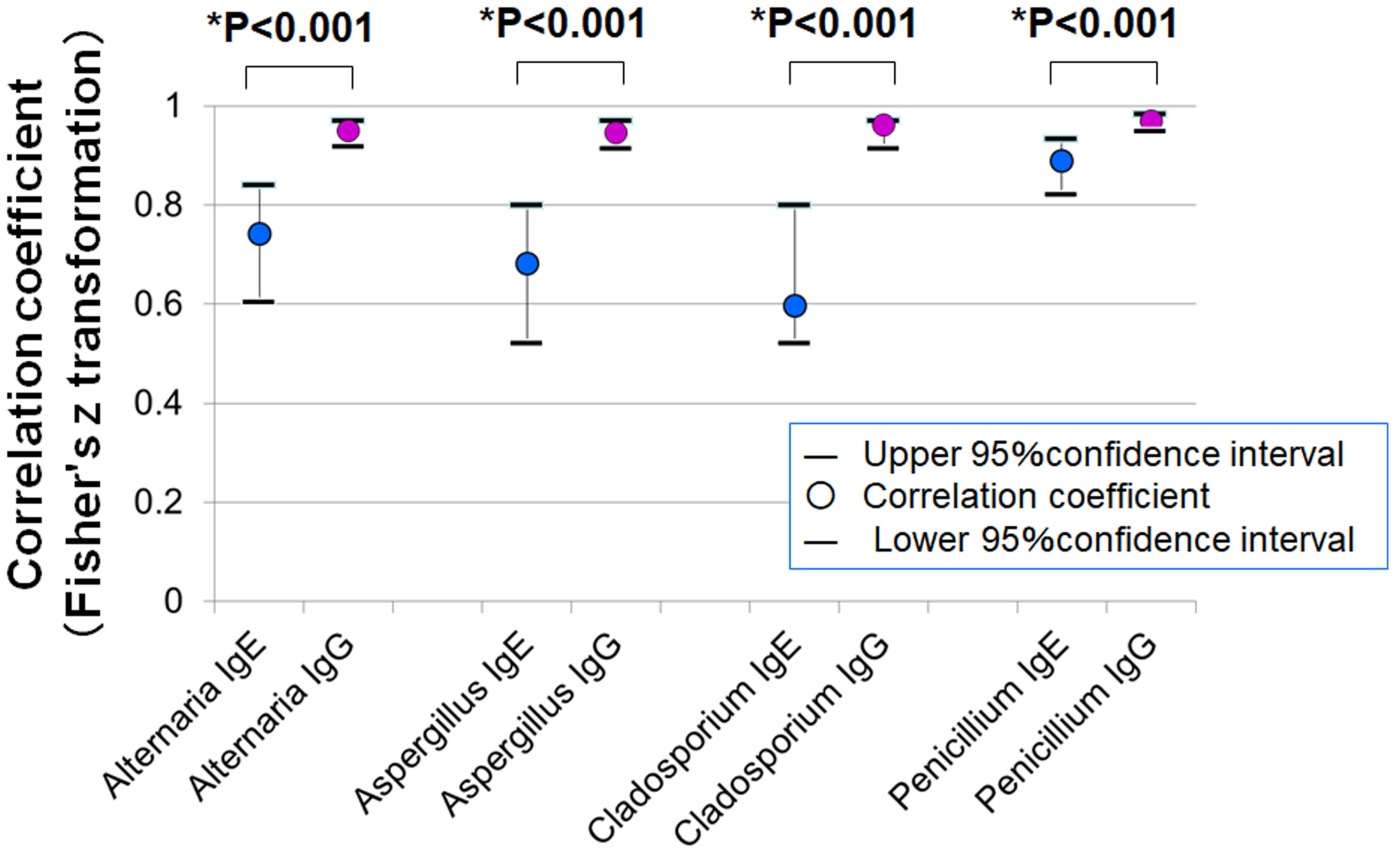
Correlations between mean wheal diameters in SPTs and serum specific IgE and IgG antibody levels for four fungal allergens. In all allergens tested (*Alternaria, Aspergillus, Cladosporium*, and *Penicillium*), IgG levels showed stronger correlations with SPT wheal diameters than IgE levels (* *p* < 0.001 for all comparisons, Fisher’s z statistics).

Figure 7 shows the correlation coefficients between average SPT wheal diameters and serum levels of specific IgE and IgG. In all four fungal allergens, the correlation with IgG was stronger than with IgE (Fisher’s z statistics):

• ***Alternaria***: *r* = 0.743 (IgE) *vs. r* = 0.949 (IgG), *p* < 0.001
• ***Aspergillus***: *r* = 0.683 *vs.* 0.947, *p* < 0.001
• ***Cladosporium***: *r* = 0.598 *vs.* 0.962, *p* < 0.001
• ***Penicillium***: *r* = 0.889 *vs.* 0.969, *p* < 0.001

### Diagnostic performance of IgE *vs.* IgG

Table 1 summarizes the diagnostic performance of serum-specific IgE and IgG for predicting SPT positivity. For *Alternaria*, *Aspergillus*, and *Cladosporium*, IgG exhibited higher sensitivity and lower false-positive rates compared to IgE, suggesting superior diagnostic accuracy.

### Association between antibody class scores and clinical severity

**Figure 8:**
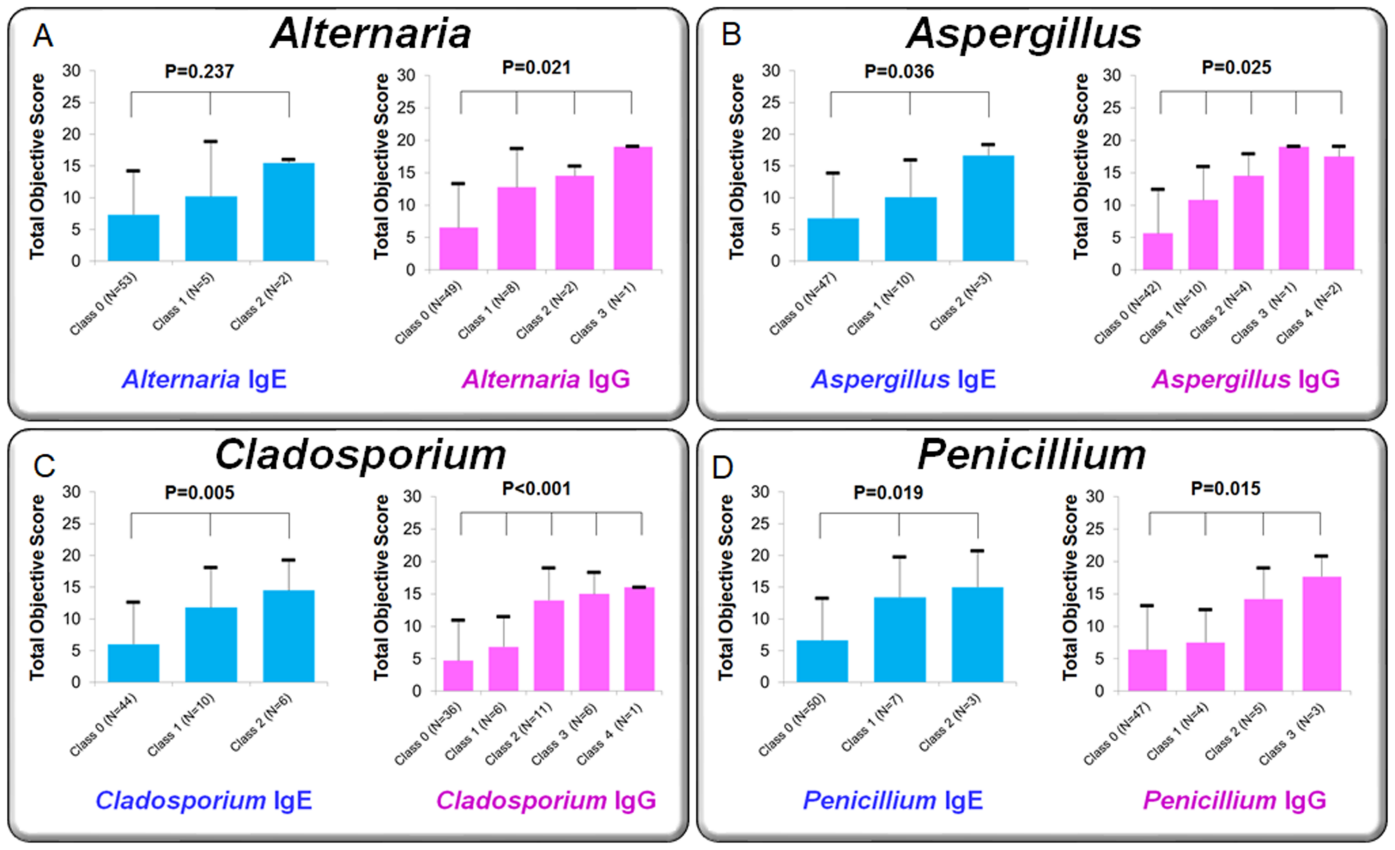
Comparison of clinical scores for allergic conjunctivitis among patient groups classified by specific IgE and IgG antibody classes for fungal allergens. Patients were divided into three groups based on IgE class (0, 1, 2) and four groups based on IgG class (0, 1, 2, 3). Panels show mean clinical scores (sum of objective findings) for each group: (A) *Alternaria*, (B) *Aspergillus*, (C) *Cladosporium*, and (D) *Penicillium*. Statistical comparisons were performed using the Kruskal–Wallis one-way analysis of variance.

Figure 8 compares mean clinical scores of allergic conjunctivitis among patient groups stratified by IgE class (0–2) and IgG class (0–3). Kruskal–Wallis tests revealed significant group differences:

• **Alternaria**: IgE *p* = 0.237; IgG *p* = 0.021
• ***Aspergillus***: IgE *p* = 0.036; IgG *p* = 0.025
• ***Cladosporium***: IgE *p* = 0.005; IgG *p* <0.001
• ***Penicillium***: IgE *p* = 0.019; IgG *p* = 0.015

In general, higher IgE or IgG class corresponded to higher clinical scores, suggesting a dose–response relationship.

### Correlation between clinical scores and antibody levels

**Figure 9:**
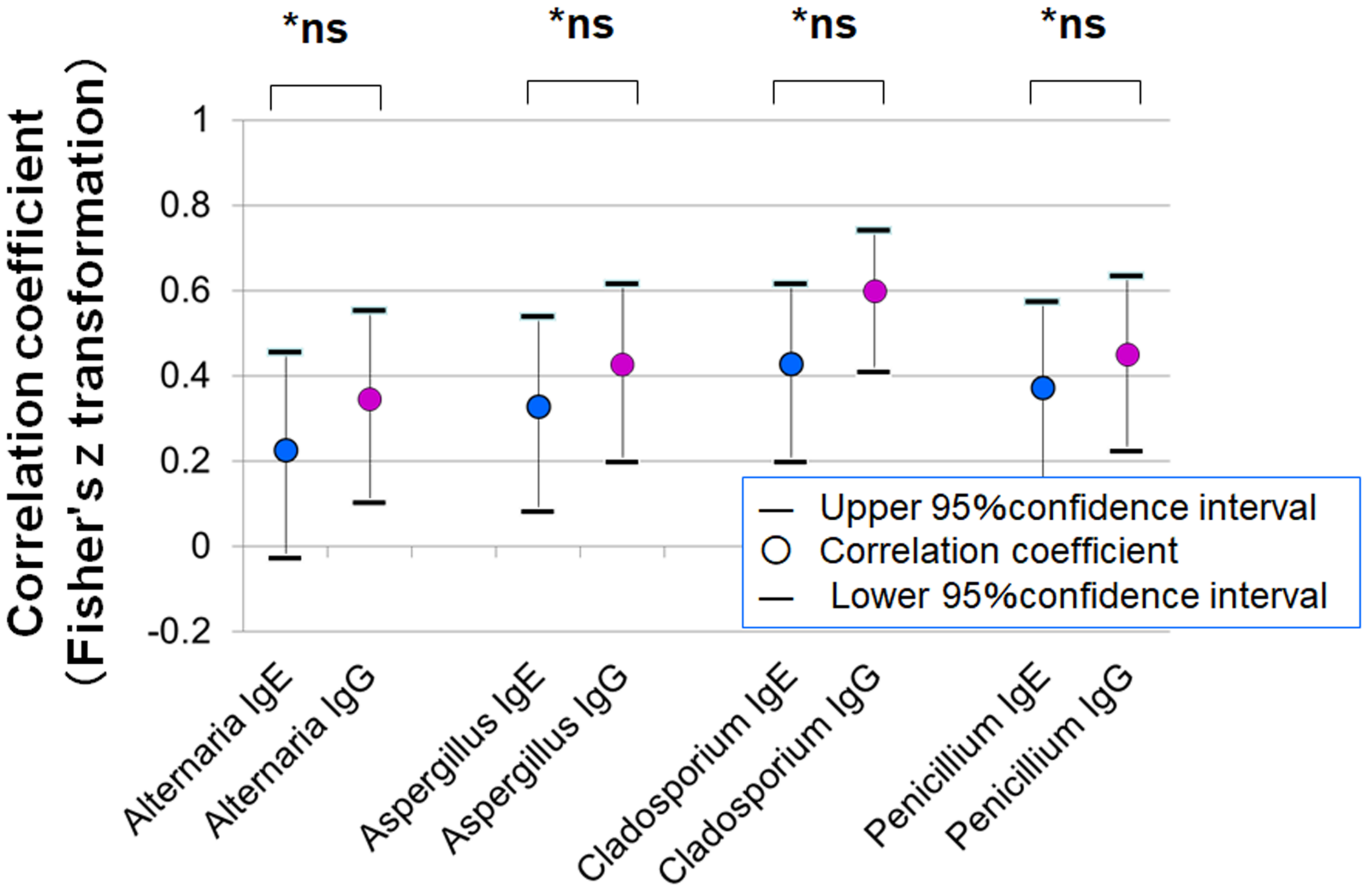
Correlation between clinical severity scores (total object score) of allergic conjunctivitis and serum-specific IgE and IgG antibody scores for fungal allergens. Fisher’s z-transformation was used to compare correlation coefficients between IgE and IgG.

Figure 9 presents the correlation between clinical severity scores and specific IgE or IgG levels. Although the correlations were not statistically significant, stronger trends were observed for IgG (Fisher’s z statistics):

• ***Alternaria***: *r* = 0.227 (IgE) *vs.* 0.346 (IgG), *p* = 0.522
• ***Aspergillus***: *r* = 0.327 *vs.* 0.428, *p* = 0.522
• ***Cladosporium***: *r* = 0.428 *vs.* 0.599, *p* = 0.222
• ***Penicillium***: *r* = 0.372 *vs.* 0.450, *p* = 0.631

### Relationship between age and IgG levels

**Figure 10:**
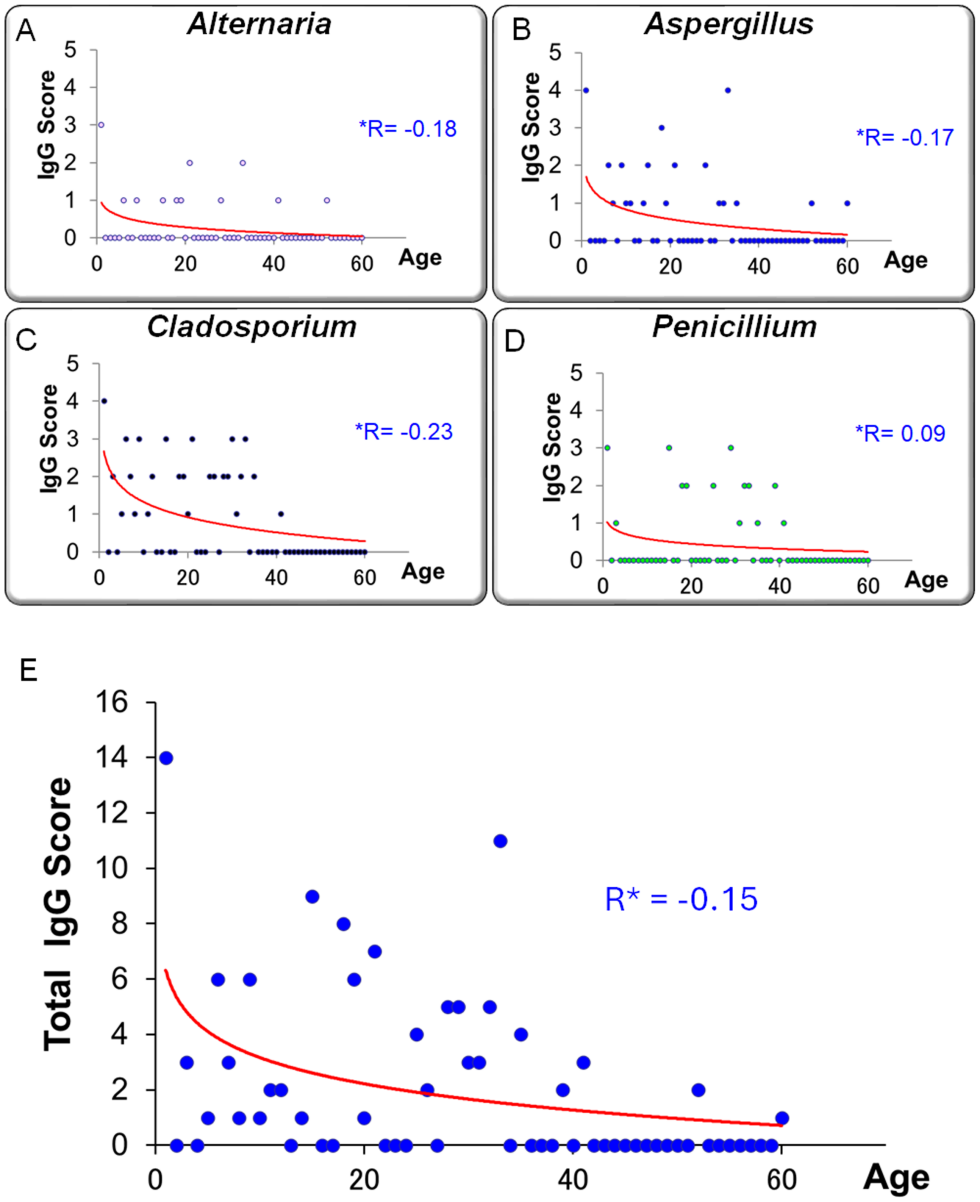
Relationship between age and serum-specific IgG scores for four fungal allergens. (A) *Alternaria*, (B) *Aspergillus*, (C) *Cladosporium*, and (D) *Penicillium*. (E) The correlation between age and the total IgG score, calculated as the sum of the four allergen-specific IgG scores. A trend toward higher IgG scores in younger individuals and lower scores in older individuals was observed, although this was not statistically significant (R *: Pearson’s correlation coefficient).

Figures 10A–10D illustrates the relationship between specific IgG scores and age for each fungal species. Figure 10E presents the correlation between total IgG score (sum of four fungi) and age. A trend toward higher IgG scores in younger individuals was observed, although this did not reach statistical significance (*r* = −0.152, *p* = 0.245, Pearson’s correlation coefficient).

## Discussion

### Summary of the study

In this study, patients with allergic conjunctivitis exhibited significantly higher positivity rates and antibody titers for specific IgE and IgG against fungal antigens (*Alternaria, Aspergillus, Cladosporium*, and *Penicillium*) compared to healthy controls. Notably, specific IgG showed a stronger correlation with SPT positivity, wheal diameter, and conjunctivitis severity scores than specific IgE. Furthermore, stratification by IgG antibody class demonstrated clearer distinctions in clinical severity scores (Fig. 8), and IgG outperformed IgE in terms of diagnostic sensitivity and lower false-positive rates in relation to SPT results.

**Table 1:** Diagnostic accuracy of serum allergen-specific IgE and IgG antibody levels for predicting skin prick test (SPT) results.

	*Alternaria*		*Aspergillus*		*Cladosporium*		*Penicillium*	
	IgE	IgG	IgE	IgG	IgE	IgG	IgE	IgG
Sensitivity	41.7%	83.3%	63.2%	89.5%	64.0%	96.0%	100.0%	100.0%
Specificity	95.8%	97.9%	97.6%	97.6%	100.0%	100.0%	100.0%	100.0%
False-positive rate	58.3%	16.7%	36.8%	10.5%	36.0%	4.0%	0.0%	0.0%
False-negative rate	4.2%	2.1%	2.4%	2.4%	0.0%	0.0%	0.0%	0.0%
Positive predictive value	71.4%	90.9%	92.3%	94.4%	100.0%	100.0%	100.0%	100.0%
Negative predictive value	86.8%	95.9%	85.1%	95.2%	79.5%	97.6%	100.0%	100.0%
Positive likelihood ratio	10.0	40.0	25.9	36.7	- (∞)	- (∞)	- (∞)	- (∞)

### Potential of IgG as a novel indicator of immune response

IgG has attracted attention as an indicator of chronic antigen exposure and immune activity, owing to its higher serum concentration and longer half-life compared to IgE. In the field of respiratory medicine, numerous studies have reported that fungus-specific IgG is significantly associated with disease severity and recurrence risk in conditions such as CRS and nasal polyposis (Hung et al., 2024; Shin et al., 2004; Pant et al., 2005; Chang et al., 2023). For example, in CRS with nasal polyps, a positive correlation has been observed between polyp severity and the amount of fungus-specific IgG, suggesting that IgG may reflect disease activity more accurately than IgE (Hung et al., 2024). Similarly, *Aspergillus fumigatus*-specific IgG has been proposed as a potential biomarker for classifying CRS into type 2 endotypes and predicting postoperative recurrence (Chang et al., 2023). In our study, serum levels of fungus-specific IgG showed significant correlations with objective clinical scores in multiple fungal allergens (Fig. 8), with trends generally stronger than those observed with IgE classes. Correlation coefficients between IgG and clinical severity scores were also consistently higher than those for IgE (Fig. 9). These results suggest that IgG may be associated with clinical manifestations in allergic conjunctivitis; however, causal relationships cannot be inferred from these correlations.

IgE is the key mediator of type I hypersensitivity reactions, associated with immediate allergic responses *via* Th2-type immune activation (Gould et al., 2003). In contrast, IgG is predominantly regulated by Th1 cells and plays a role in pathogen defense and secondary immune responses, as well as in type II and III hypersensitivity mechanisms (Akdis & Blaser, 2001). The associations observed in this study are consistent with the hypothesis that Th1-biased immune responses could contribute to the chronicity of allergic conjunctivitis, but this remains to be tested in future mechanistic studies.

Overall, these findings highlight the potential utility of comprehensive immune profiling—including measurement of specific IgG antibodies, in addition to IgE—in generating hypotheses regarding the pathophysiology of allergic conjunctivitis, particularly in the context of fungal antigens. Further studies including Receiver Operating Characteristic (ROC) analyses, optimal threshold determination, and internal validation are required to assess the diagnostic performance of IgG as a clinical biomarker.

### Superiority of IgG in correlation with SPT responses

In this study, serum levels of fungus-specific IgG antibodies against four fungal antigens (*Alternaria*, *Aspergillus*, *Cladosporium*, and *Penicillium*) showed significantly stronger correlations with wheal diameters in the SPT than those of specific IgE antibodies (Fig. 7). Fisher’s z-statistics confirmed that the difference in correlation coefficients between IgG and IgE was statistically significant for all four fungal species, suggesting that IgG may more accurately reflect SPT reactivity. Furthermore, in evaluating diagnostic performance for distinguishing SPT-positive and -negative individuals, IgG antibodies demonstrated both higher sensitivity and lower false-positive rates compared to IgE antibodies (Table 1), highlighting their potential as more reliable biomarkers. Notably, for *Cladosporium*, the correlation coefficient between IgG levels and wheal size reached an exceptionally high value of 0.962, far surpassing that of IgE (*r* = 0.598).

These findings suggest that IgG antibodies may reflect broader immune responses beyond the type I hypersensitivity reactions typically mediated by IgE. Specifically, IgG may also be involved in type III (immune complex-mediated) and type IV (delayed-type) hypersensitivity mechanisms. IgG is primarily produced under cytokine control from Th1 cells and is known to participate in immune responses characterized by chronic inflammation and tissue damage (Galli, Tsai & Piliponsky, 2008). In the conjunctiva, local IgG-mediated immune responses could be associated with chronic inflammatory signs such as hyperemia, edema, and follicular formation.

Another clinically important observation is the existence of cases that were negative for IgE but positive for IgG, indicating that fungus-related hypersensitivity may be missed if diagnostics rely solely on IgE measurements. In such instances, IgG serves as a complementary marker that can detect immune responses not captured by IgE, particularly in mild, atypical, or chronic forms of allergic conjunctivitis in which immediate-type reactions may be absent or minimal. The clinical utility of specific IgG is further supported by prior studies showing the diagnostic value of IgG antibodies against house dust mites (HDM) and animal allergens (Hales et al., 2006; Mimura et al., 2020).

Furthermore, the concurrent presence of serum IgG antibodies and positive SPT results may, in some cases, reflect repeated or high-level allergen exposure rather than a direct causal relationship between IgG production and IgE-mediated sensitization. In addition, the higher prevalence of non-eosinophilic nasal polyps in Asian populations (Hung et al., 2024) may contribute to distinct immunological patterns observed in this study.

Taken together, incorporating the measurement of specific IgG into the diagnostic process for allergic diseases may enable a more comprehensive and accurate assessment, ultimately contributing to a more personalized approach to allergy management.

### Relationship between fungus-specific IgG antibodies and age

In this study, serum levels of fungus-specific IgG antibodies exhibited a negative correlation with age. This trend was particularly evident in the total IgG score, which was calculated by summing the antibody titers against four fungal species (*Alternaria*, *Aspergillus*, *Cladosporium*, and *Penicillium*). The total score tended to be higher in younger individuals and declined progressively with age (Fig. 10; *r* = −0.152). This observation may reflect age-related changes in immune function, commonly referred to as immunosenescence.

Immunosenescence is characterized by the gradual decline of adaptive immune responses with age, leading to diminished antibody production and weakened memory responses—hallmarks of decreased IgG responsiveness (Crooke et al., 2019). Age-related impairments in B cell function, depletion of naïve T cells, and skewed T cell differentiation have been associated with poor vaccine responses and increased susceptibility to infections in older adults (Haynes & Swain, 2006; Gustafson et al., 2020). These immunological alterations may also influence the capacity to mount IgG responses against fungal antigens.

Conversely, the elevated IgG scores observed in younger individuals may be attributable to environmental exposure during early life. It has been suggested that allergen exposure in infancy and early childhood plays a critical role in shaping immune development. In particular, indoor fungi such as *Alternaria* and *Aspergillus* have been implicated as sensitizing agents for allergic diseases and asthma (Sharpe et al., 2015). Early and repeated exposure to these fungi may facilitate immune sensitization and drive higher production of specific IgG antibodies.

Importantly, fungus-specific IgG antibodies are not merely markers of sensitization; they are also indicative of repeated antigen exposure and the overall activation status of the immune system (Kang et al., 2016). Unlike IgE, which is typically involved in immediate hypersensitivity reactions, IgG—especially the IgG4 subclass—has been associated with delayed-type hypersensitivity, chronic inflammation, and immune tolerance, acting as a “blocking” antibody in certain contexts (Akdis & Akdis, 2014). Therefore, elevated fungus-specific IgG levels in younger individuals may not only reflect environmental exposure but also represent immunological profiles linked to disease susceptibility or progression.

These findings underscore the importance of considering age-related differences in immune responses when evaluating diagnostic and preventive strategies against fungal allergens. Longitudinal monitoring of fungus-specific IgG antibody levels could serve as a valuable tool for early detection of allergen sensitization, assessment of risk for disease exacerbation, and optimal timing of interventions such as environmental control or immunotherapy.

### Clinical implications and future directions

The measurement of fungus-specific IgG antibodies offers a promising approach to visualizing chronic inflammatory responses that are often difficult to detect through conventional SPT alone. As demonstrated in respiratory medicine, IgG levels have been utilized to classify disease severity, guide treatment selection, and predict recurrence risk. Similar diagnostic algorithms may be developed for allergic conjunctivitis. This study represents a foundational step toward such advancements, providing a basis for integrating IgG screening into future clinical workflows for allergic ocular disease.

It is also important to distinguish between humoral IgG-mediated immune responses and non-IgE-mediated, cell-mediated hypersensitivity mechanisms. Non-IgE-mediated immune reactivity, which can be assessed using ex vivo assays such as the Leukocyte Adherence Inhibition Test, has been shown to play a role in allergic responses to fungi such as *Alternaria alternata* and *Aspergillus fumigatus* (Olivier et al., 2023; Olivier et al., 2024). While our study focuses on IgG-mediated humoral responses, these findings highlight the complementary contribution of cell-mediated mechanisms in the pathophysiology of allergic diseases, underscoring the need for integrated approaches in line with the modern European Academy of Allergy and Clinical Immunology (EAACI) hypersensitivity classification.

Improved recognition of humoral non-IgE-mediated fungal sensitization may facilitate more accurate diagnosis and personalized management of allergic conjunctivitis. Once identified, targeted interventions such as allergen eviction and desensitization therapy could be considered to alleviate symptoms and prevent chronic disease progression.

### Limitations

Several limitations of this study should be acknowledged. First, this was a single-center study with a relatively small sample size, particularly in the control group, which included only 20 individuals. This raises the possibility of sampling bias due to regional or environmental factors. Second, because IgG responses may reflect not only allergic sensitization but also previous exposure or non-allergic immune activity, the risk of false-positive results cannot be ruled out. Thus, IgG antibody levels should not be interpreted in isolation but rather in conjunction with IgE measurements and SPT results. Third, the study lacked longitudinal data; we were unable to assess the temporal dynamics of IgG titers, such as changes before and after treatment or seasonal variations, which are important for understanding the clinical utility of IgG monitoring.

## Conclusions

This study demonstrated that fungus-specific IgG antibodies are a valuable indicator of both disease severity and fungal sensitization in patients with allergic conjunctivitis. Similar to chronic allergic diseases of the respiratory tract, IgG measurement may serve as a useful complement to traditional IgE-based diagnostics, aiding in more comprehensive assessments of disease status and treatment planning. Future research should focus on multicenter, longitudinal studies to establish standardized IgG cutoff values and clinical thresholds for improved diagnostic accuracy and individualized care.

## Supplemental Information

10.7717/peerj.20625/supp-1 Supplemental Information 1 Data (English)

10.7717/peerj.20625/supp-2 Supplemental Information 2 Protocol in Japanese

10.7717/peerj.20625/supp-3 Supplemental Information 3 Protocol in English

10.7717/peerj.20625/supp-4 Supplemental Information 4 The CONSORT PRO Reporting Guidance Checklist

10.7717/peerj.20625/supp-5 Supplemental Information 5 STROBE checklist

10.7717/peerj.20625/supp-6 Supplemental Information 6 Codebook

10.7717/peerj.20625/supp-7 Supplemental Information 7 Japanese Ophthalmic and Allergology JACQLQ (Japanese)

10.7717/peerj.20625/supp-8 Supplemental Information 8 Japanese Ophthalmic and Allergology JACQLQ (English)

## Additional Information and Declarations

### Data Availability

The following information was supplied regarding data availability: The raw data are available in the Supplemental File.

### Clinical Trial Registration

The following information was supplied regarding Clinical Trial registration: UMIN000013684.
