# Supplementary material for: Fungal-specific IgG responses in allergic conjunctivitis: comparison with IgE and immunological implications
Source: PeerJ. 2026 Jan 8;14:e20625. doi: 10.7717/peerj.20625 (PMC12790789; doi:10.7717/peerj.20625)
Supplement: Supplemental Information 7 [file peerj-14-20625-s007.pdf]

2018年　1月　12日

日本眼科アレルギー学会　ご担当者　様

三村　達哉

使用許諾のお願い

拝啓　時下ますますご清栄のこととお慶び申し上げます。

さて、貴著作物を利用させていただきたくお願い申し上げます。お手数ですが、下記内容のご確認をいただき、承認欄にご記入のうえご返送いただければ幸いです。

敬具

記

〇転載許可をお願いする図表

著　者　名：アレルギー性結膜疾患診療ガイドライン編集委員会

論　文　名：アレルギー性結膜疾患診療ガイドライン　JACQLQ

雑誌（書籍）名：日眼会誌

発行所　公益財団法人日本眼科学会

2018年　1月18日

上記の使用を許可する。

ご芳名　日本眼科アレルギー学会
