# Supplementary material for: Fungal-specific IgG responses in allergic conjunctivitis: comparison with IgE and immunological implications
Source: PeerJ. 2026 Jan 8;14:e20625. doi: 10.7717/peerj.20625 (PMC12790789; doi:10.7717/peerj.20625)
Supplement: Supplemental Information 8 [file peerj-14-20625-s008.pdf]

Jan. 12, 2018

Japan Ophthalmic Allergology Society Person in Charge

Tatsuya Mimura

Request for permission

Dear Sir, I would like to congratulate you on your continued prosperity in the coming days.

By the way, I would like to ask you to use your copyrighted work. We apologize for the inconvenience, but we would appreciate it if you could check the following contents, fill in the approval column, and return it.

Kind regards

record

○ Figures and tables for which permission to reprint are requested

Author: Editorial Committee of the Clinical Practice Guidelines for Allergic Conjunctival Diseases

Title : Clinical Practice Guidelines for Allergic Conjunctival Diseases JACQLQ

Magazine (book) name: Nichigan Journal

Publisher: The Japan Ophthalmological Society

Jan. 18, 2018

Permission is granted to use the above.

Japan Ophthalmic Allergology Society
